# Health and morbidity among those in paid work after age 64: a systematic review

**DOI:** 10.1093/eurpub/ckac131.259

**Published:** 2022-10-25

**Authors:** K Farrants, K Alexanderson, J Dervish, S Marklund

**Affiliations:** Division of Insurance Medicine, Department of Clinical Neuroscience, Karolinska Institutet, Stockholm, Sweden

## Abstract

**Background:**

Despite the increase of labour market participation at older ages, very little is known about health and morbidity among those who remain in a paid work after age 64. The aim was to systematically review the scientific knowledge on health and morbidity among people aged above 64 years who are in paid work.

**Methods:**

A systematic literature review of studies published in English in scientific journals in 2014-2020. We identified 18,972 unique publications, of which 66 were deemed relevant by at least two independent researchers. Quality judgements and data extraction were done by at least two independent researchers according to pre-specified templates.

**Results:**

There was a great heterogeneity in the included studies regarding study design, included populations (both size and type), exposures, outcomes, covariates, measures, and analytical methods. Few were assessed as having high quality. Most studies (95%) were from OECD countries and results were about men to a greater extent than women. 42 of the 66 studies had results indicating that being in paid work after age 64 was associated with good health and less morbidity. Six studies presented at least one result showing the opposite; those in paid work had worse health than those not, while 21 studies presented at least one result showing that there were no health/morbidity differences between those in paid work and who were not. Only one study presented results regarding mortality. Many aspects had not been studied at all, or only in one or two studies.

**Conclusions:**

Many studies had results indicating that those who were in paid work >64 had better health/less morbidity than those who were not, however, there was a great variety in the results. There are surprisingly few studies about health/morbidity among people in paid work after age 64, and those published are heterogeneous: it is thus not possible to draw conclusions regarding scientific evidence based on the currently existing studies.

**Key messages:**

• Both study designs and results were very heterogenous in the 66 studies that presented results on health or morbidity among people in paid work after age 64.

• More and better studies are needed as well as greater clarity regarding study designs, populations, measures, analytical methods and definitions of central concepts such as work, health and morbidity.

